# Dietitians as Food Systems Changemakers: The Path to Developing a Resilient, Equitable, Healthy and Connected Food System in the Illawarra Shoalhaven Region of Australia

**DOI:** 10.1111/jhn.70264

**Published:** 2026-05-06

**Authors:** Karen Charlton, Suzanne Pickles, Alemayehu Digssie Gebremariam, Selena Stevens, Anita Stefoska‐Needham, Katherine Kent

**Affiliations:** ^1^ School of Health Science University of Newcastle Callaghan New South Wales Australia; ^2^ Nutrition and Metabolic Health Program, Hunter Medical and Health Research Institute New Lambton New South Wales Australia; ^3^ School of Medical, Indigenous and Health Sciences University of Wollongong Wollongong New South Wales Australia; ^4^ Regional Development Australia Illawarra Shoalhaven Wollongong New South Wales Australia; ^5^ School of Health Sciences University of New South Wales Kensington New South Wales Australia

**Keywords:** changemaker, collective impact, design‐led, dietitian, regional food system, systems thinking

## Abstract

**Background:**

Contemporary food systems contribute to climate change and influence food security, diet quality, equity and regional resilience. Addressing these interconnected challenges requires coordinated, place‐based actions across the entire food system, with dietitians and nutrition professionals increasingly recognised as key system actors.

**Objective:**

To describe a dietitian‐led, systems‐thinking approach used to inform the development of a regional food strategy in New South Wales, Australia and to identify opportunities for dietitians and nutrition professionals in food system change across health, equity and environmental sustainability domains.

**Methods:**

Using a socioecological model of health promotion and a collective impact methodology, a 2‐year evidence‐building and co‐design programme of work was undertaken. Mixed methods were used across Ottawa Charter action areas: Building Healthy Public Policy; Creating a Supportive Environment; Developing Personal Skills; and Strengthening Community Action. Activities included diet affordability analysis, food environment and production mapping, community surveys, social network analysis, pilot skills‐building initiatives and cross‐sector stakeholder engagement.

**Results:**

The programme generated a coordinated regional evidence base on food security, food environments and local food systems, which informed the establishment and governance structure of a cross‐sector Food Futures Taskforce and the co‐design of a regional Food Charter and Action Plan with defined priorities and responsibilities. Findings highlight the central role of dietitians as knowledge translators, equity advocates and facilitators of systems change.

**Conclusion:**

This case study demonstrates how dietitians can operationalise systems thinking to catalyse regional food system governance and transformation. The approach offers a transferable model for integrating research, policy and practice to advance healthy, equitable and sustainable food systems.

## Summary

- This case study demonstrates how dietitians can operationalise systems thinking in practice to catalyse regional food system governance, moving beyond traditional clinical or educational roles.
- A dietitian‐led, evidence‐driven, collective impact approach successfully integrated mixed‐methods data (diet affordability, food environments, civic food assets, community voices) into shared governance structures, including a regional Food Futures Taskforce, Food Charter and Action Plan.
- Findings illustrate dietitians' roles as knowledge translators, equity advocates, boundary spanners and facilitators of collective action, bridging health, planning, community and economic development sectors.
- The study provides a transferable model for integrating research, policy and practice to advance healthy, equitable and sustainable food systems at municipal and regional levels.

## 1 Introduction

Transforming contemporary food systems is now widely recognised as essential for advancing public health, equity and planetary sustainability [1, 2]. Recent global syntheses argue that food systems sit at the nexus of human health, climate, biodiversity and social justice, and that achieving healthy diets within planetary boundaries requires coordinated, multi‐level action that extends far beyond individual behaviour change.

The updated EAT–Lancet Commission (2025) reframes these challenges in light of post‐pandemic shocks, geopolitical instability and escalating food prices, calling for just, sustainable, healthy food systems and identifying solution areas that hinge on cross‐sector governance and place‐based implementation [3]. In parallel, city and regional governments are consolidating their role as implementers of food policy via instruments such as the Milan Urban Food Policy Pact (MUFPP) and its monitoring framework, which provide practical indicators and governance guidance to help localities design, track and improve integrated food strategies [4].

Within this evolving policy landscape, dietitians and nutrition professionals are increasingly positioned as system actors whose competencies span evidence synthesis, food‐environment assessment, procurement and menu standards, communication and collaborative leadership [5, 6, 7, 8, 9]. International standards from the Academy of Nutrition and Dietetics specify performance indicators for sustainable, resilient and healthy food and water systems across competence levels, signalling expectations for practice that link nutrition outcomes to environmental and social domains [6]. Likewise, Dietitians of Canada articulate roles for dietitians across professional practice, management, population health, research and advocacy, advancing a planetary‐health perspective that embeds sustainability into routine dietetic work [9]. An important but historically under‐engaged contribution to this literature is civic dietetics, which builds on Lyson's concept of civic agriculture to posit dietetics as a profession that can strengthen local food democracy by connecting producers, institutions and communities through values‐driven, place‐based practice [10]. Wilkins [10] argues that dietitians can help further civic agriculture and sustainable food systems by integrating sustainability and community engagement into core roles, while recognising organisational constraints that have historically narrowed professional practice. Contemporary work on collaborative leadership further evidences the potential of dietitians to drive sustainability within health systems. Highlighted examples include food‐waste reduction, supply‐chain improvement and the management of tensions between providing health and environmental advice, all of which are being driven by the need for allied‐health leadership required to meet net‐zero targets within health systems [11]. Together, these perspectives reframe dietitians not only as clinical educators but also as knowledge translators, equity champions and boundary spanners who can convene cross‐sector coalitions, translate research into policy and co‐design structural change within local food environments [6, 9, 12].

Despite these advances, empirical accounts describing how dietitians operationalise systems thinking to catalyse regional food governance change, by linking evidence generation, stakeholder networks and co‐designed charters into actionable policy and practice, remain limited. This gap is particularly salient for municipal and regional settings where food‐system responsibilities are dispersed across planning, health, education, welfare and economic development portfolios, and where frameworks like MUFPP emphasise the need for shared measurement, inclusive governance, ongoing monitoring and community engagement.

In the Illawarra Shoalhaven region of New South Wales (NSW), Australia, we established a multi‐year, dietitian‐led programme to strengthen regional food production and consumption using systems There is thinking, design‐led approaches [13] and collective‐impact principles [14]. Guided by a socioecological model and four action areas of the Ottawa Charter [15] (Building healthy public policy, Creating supportive environments, Strengthening community action and Developing personal skills), the first 2 years (2023–2025) of the project focused on building a shared evidence base (i.e., diet affordability; food environments; local production assets; community survey; social network analysis [SNA]), translating findings into accessible outputs and convening cross‐sector governance through a Food Futures Taskforce [16] (Taskforce), leading to a co‐designed Food Charter and an accompanying 3‐year Action Plan.

This paper presents a case study of a dietitian‐led approach to regional food‐system transformation. Rather than reporting final intervention outcomes, we examine how systems thinking was operationalised through evidence generation, collective‐impact governance and design‐led processes to inform co‐ordinated action. Situating our work within civic dietetics and collaborative‐leadership literature [10], we provide transferable lessons on the roles that dietitians and nutrition professionals can play in catalysing food‐system change across health, equity and sustainability domains. This also offers a replicable model for other regions implementing MUFPP‐aligned strategies.

## 2 Materials and Methods

### 2.1 Context

The Illawarra Shoalhaven region of NSW begins 80 km south of Sydney and has a growing community and nationally significant assets. The unique combination of mountains, bushland and sea sweeps across a total land area of 5656 square kilometres. The region includes four local government areas: Wollongong, Shellharbour, Kiama and Shoalhaven. Recognised for its contribution to manufacturing (notably heavy metallurgy, coal mining and steelmaking), logistics, healthcare and education, the region has a GRP of $33 Billion and represents the third largest economy in NSW. Built on a long legacy of industry, the region continues to be a place of innovation, growth and economic diversity, with emerging sectors such as clean energy, advanced manufacturing, tourism, agribusiness and creative industries complementing traditional heavy industries. Today, the region includes more than 427,300 people, with this number expected to grow to 463,150 by 2036 [17]. The NSW Department of Planning, Housing and Infrastructure identifies the Illawarra Shoalhaven as one of the fastest‐growing regions in the state, with total population growth of approximately 36% by 2041 under current assumptions [18].

At a state and local level, planning mechanisms effecting the Illawarra Shoalhaven region typically prioritise housing and infrastructure over protection of prime farmland. These frameworks are often shaped through vested interests between policymakers and stakeholders within the property development sector, resulting in land‐use regulations, such as zoning ordinances, that constrain agricultural activities [19, 20]. Unlike urban centres in many high‐income nations, such as the Greater Toronto and Hamilton area green belt area in Southern Ontario, metropolitan areas in NSW typically lack designated green belt zones intended to safeguard peri‐urban agricultural landscapes [21]. Within this context, the Illawarra Shoalhaven catchment possesses a moderate extent of land with suitable agricultural capability and inherent soil fertility conducive to food production. As of 2020, approximately 18% of land in the region was zoned for rural agricultural use, with the majority (76%) comprising small allotments under 20 hectares, rendering them largely unsuitable for broadacre cropping or livestock enterprises [20].

Despite the fragmentation of agricultural land in the Illawarra Shoalhaven region, a limited number of dairy and beef operations persist. However, the absence of local processing infrastructure, such as abattoirs and large‐scale dairies, has necessitated the external transport of produce for processing and distribution, thereby diminishing the region's capacity to support a cohesive local food system [22]. In contrast, small‐scale urban and peri‐urban agricultural enterprises contribute meaningfully to local food networks, cultivating a diverse array of products including vegetables, fruits, mushrooms and honey [23]. Nevertheless, these operations are not captured within publicly accessible agricultural data sets, indicating a significant gap in existing data collection and reporting mechanisms [20].

### 2.2 Collective Impact Methodology and Design‐Led Approaches

The term ‘wicked problem’, first coined by Rittel and Webber, describes complex societal problems that resist traditional linear problem‐solving approaches because of multiple interacting causes, competing values and uncertain outcomes [24]. This project proposes that the contemporary food system poses a ‘wicked problem’ [24] in terms of its adverse impact on climate change, inequitable access to healthy and sustainable foods, and political and economic drivers that favour ‘big food’ instead of supporting local food system approaches. Complex problems occur across traditional sector boundaries and are shaped by interacting social, economic and environmental determinants, requiring the input of a diverse range of stakeholders and multi‐faceted interventions. Because these dynamics are experienced and enacted locally, such challenges are often addressed through a *place‐based approach*, which focuses on coordinated action within a defined geographic area and prioritises changes to social and physical environments rather than individual behaviour [25].

**Figure 1:**
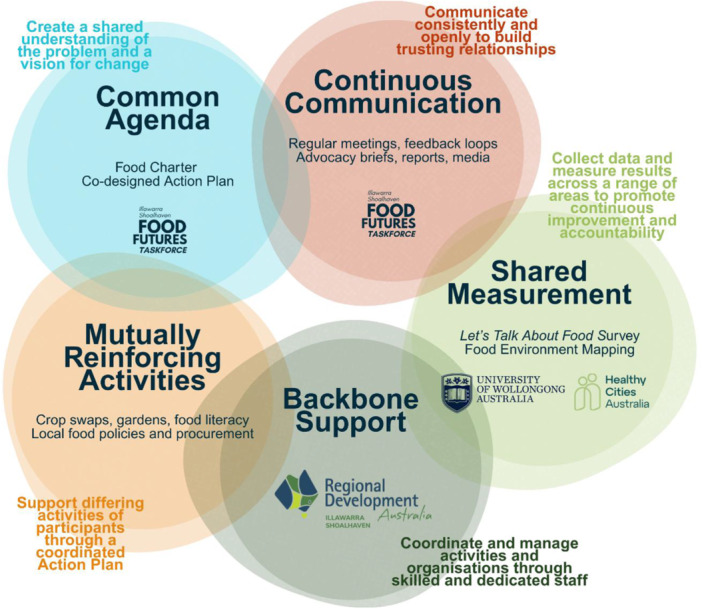
Five aspects of Collective Impact Methodology, as applied to the Illawarra Shoalhaven Food Systems project (26).

The project utilises Collective Impact methodology [26] which is a structured, cross‐sector approach to tackling complex social challenges. It relies on five key conditions: a common agenda, shared measurement systems, mutually reinforcing activities, continuous communication and the presence of a backbone organisation to coordinate and sustain efforts (see Figure 1). A well‐documented example of Collective Impact applied to local food system development is the Farm‐to‐Plate (F2P) Investment Program in Vermont, USA. Emerging from a coordinated, cross‐sector Collective Impact approach, the programme has supported the development of a regionally embedded agribusiness and food systems model [27].

In Australia, the Collective Impact approach is being increasingly used to bring about population‐level change on complex social issues that have multiple and intersecting causes such as homelessness [28], poverty [29], climate change [30] and childhood obesity. Despite Australia producing sufficient food to feed around twice its population (approximately 50–60 million people), a large share of agricultural output is exported [31]. Paradoxically, this national abundance co‐exists with rising food insecurity, with around one in eight Australian households experiencing food insecurity in recent years. A design thinking methodology was applied to support the collective impact approach within a complex regional food system context. Design thinking is an iterative, human‐centred approach to problem solving that is well suited to addressing complex or ‘wicked’ challenges [32], as it prioritises lived experience, collaboration and adaptive learning. In the dietitian‐led programme, the approach was used to facilitate shared understanding among diverse stakeholders and to generate potential actions that were responsive to local needs while remaining feasible within existing technological and economic constraints [13, 33]. The process focused on aligning three interrelated considerations, including ‘desirability’ from the perspective of community members and stakeholders, ‘feasibility’ within the technical and organisational context of the regional food system, and ‘viability’ in relation to available resources and longer‐term sustainability [34]. Attention to this intersection was central to guiding decision‐making throughout the co‐design process and to ensuring that proposed actions were both contextually appropriate and implementable, as guided by previous research by Stefoska‐Needham et al. [13]. Typically, the most valuable solution or idea is one that meets all three criteria.

The design thinking process was operationalised through repeated cycles of stakeholder engagement, collective sense‐making and refinement. Key system actors, including community members, practitioners and organisational representatives, were actively involved as co‐design participants rather than as consultees. Iterative cycles of idea generation and feedback enabled emerging insights to be tested against real‐world constraints, with learning from each cycle informing subsequent stages of the process. The approach followed five inter‐related phases commonly described in design thinking practice as follows: empathise, define, ideate, prototype and test [32, 35]. The empathise phase involved direct engagement with stakeholders to explore experiences, priorities and perceived challenges within the regional food system, supporting a shared understanding of the problem space from multiple perspectives [36]. Insights generated during this phase informed the define phase, in which key needs and system challenges were synthesised and articulated to guide collective action. The ideation phase focused on the generation of potential strategies and interventions (or solutions) through collaborative brainstorming and exploration of system‐level leverage points. Prototyping involved the development of tangible representations of proposed actions, such as draft governance structures, frameworks or pilot initiatives, which could be examined and refined in practice. The test phase incorporated structured feedback and reflection to assess alignment with stakeholder needs and system conditions, enabling ongoing adaptation prior to broader implementation.

The design thinking approach requires reflexivity to be embedded throughout the research process. This involves creating deliberative processes for researchers, practitioners and community members to continually examine their own values, roles, assumptions and power dynamics. Reflexivity involves both active individual and collective critical reflection and is considered an important capacity for researchers from different disciplines to address the resulting ethical and practical challenges [37]. Reflexivity deepens learning, equity and adaptability—all of which are essential for transformative work in complex systems.

SNA is a methodological approach used to examine the structure and patterns of relationships among actors (e.g., individuals, organisations or institutions) in order to understand how connections, positions and flows of information or resources influence behaviour and outcomes within a system [38]. SNA was used to identify the positioning of dietitians and nutritionists identified as active within the local food network and to better understand the attributes of those individuals so that opportunities and constraints for future strategic involvement could be recognised.

### 2.3 Framework and Vision for a Healthy, Equitable and Sustainable Local Food System

**Figure 2:**
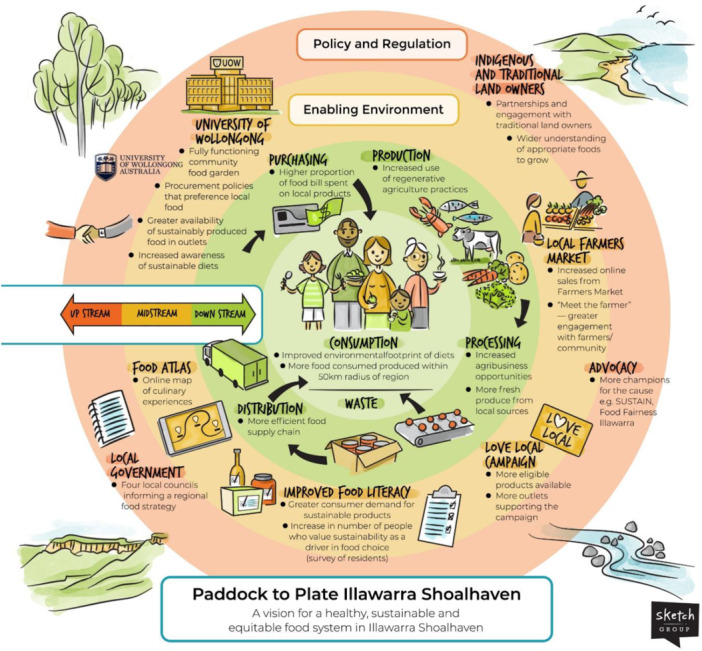
Vision for development of a healthy, sustainable and equitable regional food system using collective impact methodology.

Figure 2 represents the project's vision which is to create a food system in the Illawarra Shoalhaven region that emphasises local economic support, sustainable food production practices, community connection to food origins, equitable access and awareness of food, and reduction of environmental impacts. The visual flow moves from upstream (policy and regulation to set the governance and rules for a sustainable food system) through midstream (creating enabling environments) to downstream (changes in components of the food system that ultimately result in individual behaviour change). The framework emphasises cross‐sector engagement, environmental responsibility and community participation across all phases.

The stages of the food supply chain were included, with objectives provided for each, namely: production: increased use of regenerative agriculture practices; processing: more agribusiness opportunities and greater volume of fresh, local produce processed regionally; distribution: a more efficient and sustainable food supply chain; purchasing: a higher proportion of food spending goes to local products and greater engagement between producers and community; consumption: improved environmental footprint of diets and more food consumed that is produced within a 50 km radius; waste**:** reduced waste through efficient systems including resource circularity and reuse.

### 2.4 Data and Evidence‐Building Programme

**Figure 3:**
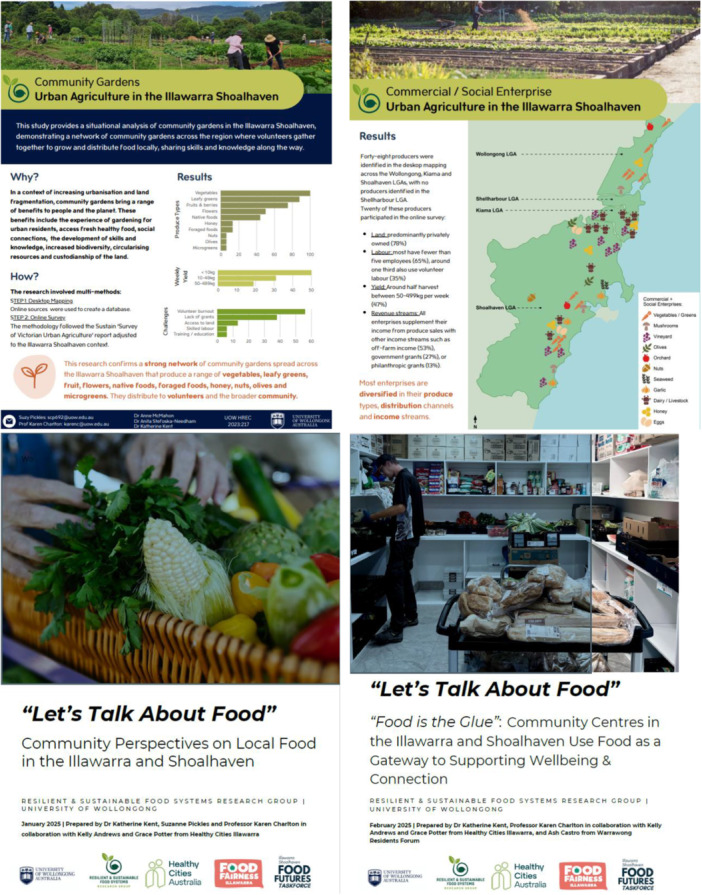
Examples of evidence briefs developed for Advocacy and to Strengthen Community Action.

A 2‐year evidence‐building programme established the foundation for regional systems transformation in the Illawarra Shoalhaven region. This programme aimed to generate locally relevant data on food environments, affordability, food insecurity and community food initiatives to inform the development of the Illawarra Shoalhaven Taskforce and Food Charter (see Figure 3). Mixed‐methods approaches were employed to capture the complexity of the regional food system, including quantitative mapping and survey data, participatory pilot studies and qualitative stakeholder engagement. Together, these studies provided the shared measurement framework for subsequent governance and implementation activities.

## 3 Results

Activities conducted in the evidence‐building and co‐design programme of work are summarised according to the four targeted Ottawa Charter action areas, and identification of the resulting systems‐change input with specific learnings for dietitians identified in each case.

### 3.1 Ottawa Action 1: Building Healthy Public Policy

Using the validated Healthy Diets ASAP protocol [39], diet cost analyses showed that recommended (i.e., healthy) diets were, on average, 16%–20% less expensive than current diets, however, they remained unaffordable for low‐income households, requiring more than 30% of disposable household income, indicating structural barriers to healthy eating [39, 40, 41]. This clearly points to structural economic, not behavioural, barriers to healthy eating faced by disadvantaged households that includes, but is not limited to, inadequate safety nets and high cost of housing. When combined with regionally specific food insecurity data from the community‐wide *Let's Talk About Food* survey (38% experiencing any food insecurity; 19% moderate; 12% severe, with higher prevalence in households with children), and qualitative accounts of price‐related distress and dietary compromise, the evidence formed a policy‐ready case for action on food affordability and social protection [42, 43, 44, 45].

Systems‐change input and learnings: Quantified affordability constraints and high food insecurity burden provided a shared problem definition for cross‐sector partners and justified policy levers (e.g., affordable food access initiatives, social safety‐net advocacy). Given the sociopolitical environment of elevated food costs and the data collection phase spanning local government elections, evidence briefs accompanied by media releases were orchestrated to be as timeous and impactful as possible. For dietitians, these findings highlight the importance of moving beyond individual dietary counselling to systematically documenting affordability constraints and elevating these data within policy and planning processes. These findings were used to brief local government and regional development partners, informing the terms of reference and priorities of the regional Taskforce (see Governance results below).

### 3.2 Ottawa Action 2: Creating Supportive Environments

Retail mapping identified 1924 outlets across the region, with only 15% classified as healthy, yielding a 1:6 healthy‐to‐unhealthy ratio, and demonstrating higher unhealthy outlet density in low‐socioeconomic areas [46]. These findings highlighted the structural nature of local food environments and the limits of individual behaviour‐change approaches in settings dominated by obesogenic retail exposure. The term obesogenic refers to environments that promote excessive energy intake and sedentary behaviour, thereby increasing the risk of obesity at the population level. In parallel, mapping of community gardens and peri‐urban producers documented under‐recognised civic assets that contribute to local food supply, food literacy, environmental sustainability and social connection [47, 48]. These alternative food networks enhance community resilience and sustainability by supplementing household diets, reducing food miles and fostering social connectedness. For dietitians, this work demonstrates the value of pairing deficit‐focused analyses (e.g., unhealthy retail density) with asset‐based mapping to create a more balanced and actionable narrative for change. By generating spatially explicit evidence, dietitians were able to shift conversations with planners and policymakers away from education‐only solutions towards upstream levers such as land‐use planning, zoning and retail mix regulation. At the same time, documenting community food assets provided practical entry points for strengthening supportive environments through partnerships with community gardens, crop swaps, food cooperatives and local producers.

Systems‐change input and learnings: The juxtaposition of obesogenic retail structures with enabling civic food assets provided a compelling, place‐based evidence base for the regional Taskforce. This enabled dietitians to advocate simultaneously for regulatory action (e.g., planning and zoning requirements to limit unhealthy outlet proliferation) and for investment in community‐led food initiatives that support access, sustainability and social cohesion. A key learning for dietitians is the importance of working across sectors, particularly with urban planning, local government and community organisations, and of using visual and spatial data to make food environment inequities visible and policy‐relevant. Through this approach, dietitians moved beyond traditional service delivery roles to act as system connectors, translating local data into practical strategies for creating healthier, more sustainable food environments.

### 3.3 Ottawa Action 3: Strengthening Community Action

SNA identified limited integration between local dietitians and nutritionists with other key stakeholders including local government, and civil society organisations, indicating coordination gaps. This study found that by increasing their participation in collaborative food‐based initiatives, nutritionists and dietitians could broker connections between the health profession and the local community, helping to foster skills and knowledge about food and farming, increasing their visibility and influence within the local food network and building their own understanding of place‐based food and farming. Complementary studies of community centres and food‐relief networks demonstrated the scale, scope and strain within charitable food systems, and the centrality of food as a gateway to connection and care in community centres [49, 50].

A shared measurement framework was established through mixed methods: geospatial mapping of food retail environments and civic assets; diet affordability analyses and population food insecurity estimates; participatory pilot studies; and qualitative stakeholder engagement. These data created a common evidence base that have enabled prioritisation of actions and a mechanism to track progress over time.

**Figure 4:**
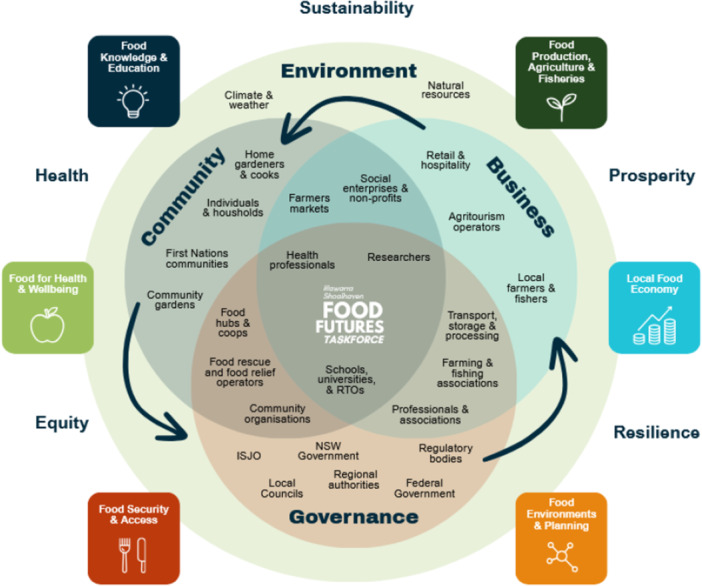
Cross‐sector collaboration of the Illawarra Shoalhaven Food Futures Taskforce with community, business and governance.

**Figure 5:**
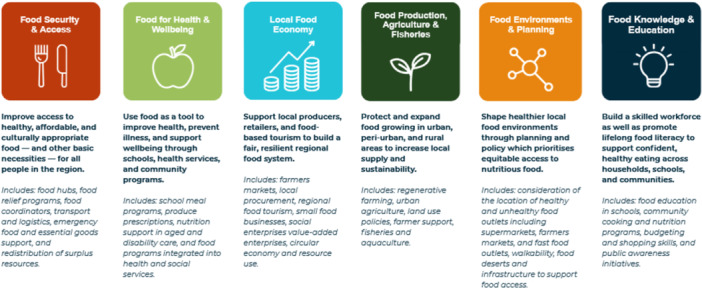
Six evidence‐informed key action areas for transformation of the Illawarra Shoalhaven regional food system (16).

Central to strengthening community action and providing agency to under‐represented groups such as civil society organisations, a deliberative, multi‐channel research translation strategy was implemented. Evidence generated through community surveys, food environment audits and dietary affordability studies were disseminated well beyond academic settings via coordinated media engagement, advocacy briefs and co‐developed community reports. Partnership‐driven dissemination has been central to this approach, through close collaboration with community organisations and networks such as Healthy Cities Australia (HCA), Food Fairness Illawarra coalition and the Warrawong Residents Forum, enabled data collection and interpretation to be embedded within ongoing community programmes. Publicly accessible evidence briefs (i.e., short, easy‐to‐read summaries released as data were analysed) served as a core dissemination mechanism (see Table S1 for outputs and metrics as of 28/11/2025). Digital engagement was strongest through the HCA/Food Fairness Illawarra coalition website, supported by a targeted media campaign that reached over 220,900 people and generated 840 link clicks during recruitment for the ‘Let's Talk About Food’ survey. Survey findings are housed on the University of Wollongong website as well as the HCA and Food Fairness Illawarra websites and have been circulated widely through social media channels. This demonstrates the value of university–non‐profit partnerships in making research accessible to the communities it represents. To date, the research has been featured in more than 18 media stories across print, radio and digital outlets, helping elevate food insecurity as a recognised health and social issue across the region. Practitioner‐ and policy‐focused outputs have further translated evidence into actionable insights, highlighting trends in household food insecurity, the role of community centres and the contributions of local food relief networks (see Figures 4 and 5 for examples). These community‐friendly reports have been downloaded approximately 3167 times.

Advocacy efforts have extended the programme's influence into government processes, with findings incorporated into two formal submissions: the Feeding Australia National Food Security Discussion Paper and Wollongong City Council's 2026 Draft Economic Development Strategy. Collectively, these activities demonstrate a systematic and sustained approach to research translation, using evidence not only to describe regional food insecurity but to catalyse coordinated action across communication, service and policy domains.

Systems‐change input and learnings: Evidence‐driven insights to community needs justified a coalition‐building focus for governance and policy/practice integration (e.g., linking food relief with prevention, procurement and local supply).

The community‐action activities described here align with the Dietitians Australia National Competency Standards [51], particularly collaboration with stakeholders, influencing population health, and critical thinking/evidence integration, and illustrate systems‐oriented professional practice. They also reflect contemporary expectations embedded in the Accreditation Standards for Dietetics Education Programs [52], with implications for curriculum design (governance, geospatial mapping methods, reflexivity) and innovative work‐integrated opportunities.

### 3.4 Ottawa Action 4: Developing Personal Skills

University‐based experiential programmes, including the *Food Warriors* gardening programme, *Veggie Box* trial and *Farmwall* education programme [52, 53], together with qualitative research involving barriers to food security in international students [54], provided proof‐of‐concept evidence for improvements in food literacy, increased access to fresh produce and enhanced confidence in food skills. These initiatives also highlighted persistent structural constraints within tertiary settings, particularly the need for culturally appropriate and affordable food supports for diverse student populations, including international students experiencing financial and food access pressures.

For dietitians, these findings reinforce the value of experiential, practice‐based learning approaches that go beyond didactic nutrition education. Hands‐on programmes that integrate growing, sourcing and preparing food enabled participants to build practical skills, strengthen food agency and reconnect dietary guidance with lived experience. Importantly, the qualitative insights underscored that food literacy interventions must be culturally responsive and financially realistic to be effective, highlighting dietitians' role in co‐designing programmes with students rather than assuming a one‐size‐fits‐all model of healthy eating.

Systems change input: These initiatives operated as deliberatively designed downstream levers within a whole‐of‐system strategy, translating abstract concepts of food literacy, sustainability and equity into lived, practical experience for students. By embedding experiential learning within campus food infrastructure and institutional partnerships, the programmes helped normalise food skills as a shared responsibility of the university environment rather than an individual deficit. For dietitians, the key systems insight is that developing personal skills can generate feedback loops that influence broader system settings. Evidence from these programmes was used to justify changes to campus‐level food access schemes, inform procurement discussions around fresh and local produce, and strengthen the case for investing in visible food infrastructure such as gardens and edible landscapes. Collectively, this demonstrates that when dietitians design experiential, culturally responsive programmes that are institutionally embedded, personal skill development becomes a catalyst for wider food system alignment rather than a standalone behaviour‐change intervention.

### 3.5 Developing a Governance Model for the Taskforce

Findings from the data collection phase (each individual project received approval from the University of Wollongong Human Research Ethics Committee approval) directly informed the establishment of the Taskforce as a central governance mechanism for regional food system transformation. Consistent with the collaborative and design‐led nature of the project, the Taskforce adopted an explicitly equitable approach to governance. This was operationalised through the intentional embedding of reflexivity in relationship‐building processes, whereby members were encouraged to critically examine their own values, roles, assumptions and positions of power. This reflexive practice fostered individual and collective accountability, an essential condition for transformative action in complex, multi‐actor systems.

To support learning‐in‐action, feedback loops (adapted from business and systems theory) were embedded throughout the Taskforce process. These loops functioned as a form of continuous evaluation, ensuring that insights generated through pilot initiatives, stakeholder engagement and community consultations were systematically fed back into subsequent phases of design‐led thinking. The iterative governance approach strengthened collective ownership, enhanced transparency, and improved the Taskforce's capacity to respond to emerging system dynamics in real time.

Despite strong engagement across sectors, several governance tensions emerged during the Taskforce's establishment. In particular, ambiguity around ownership versus responsibility for action periodically slowed progress, reflecting the inherently distributed nature of food system work across multiple sectors and levels of governance. The development of the Food Charter progressed more slowly than anticipated, underscoring the time required to build trust, acknowledge historical work in the region prior to the taskforce being established, agree on shared language and secure commitment among diverse stakeholders. The process was also reliant on a small group of highly engaged participants. While this core group was critical in maintaining momentum, it raised concerns regarding capacity, shared motives and desired outcomes, representativeness and succession.

In order to ensure that community perspectives were systematically incorporated into the accompanying Action Plan, three complementary strategies were employed: (1) an online survey of key food system stakeholders to identify regional priorities related to food security, health, equity and sustainability; (2) targeted online focus groups to explore perceived barriers and enablers in greater depth; and (3) co‐design of a draft Action Plan through a World Café–style workshop. To safeguard the authenticity of this engagement and minimise bias arising from pre‐existing academic‐community relationships, these three components were independently facilitated by the not‐for‐profit organisation Sustain (The Australian Food Network), thereby strengthening the legitimacy and inclusiveness of the governance process.

## 4 Discussion

This practice‐informed case study demonstrates how a dietitian‐led, evidence‐driven, collective‐impact approach operationalised systems thinking to move from empirical inputs (affordability, food environments, civic assets, lived experience) to a regional governance mechanism to bring about change (Taskforce), a shared agenda (Food Charter), and an Action Plan that aligns policy and practice. The contribution of this paper is process‐level whereby we elucidate mechanisms by which dietitians and nutritionists can catalyse municipal/regional policy action.

Our findings extend the civic dietetics proposition by showing dietitians functioning as boundary spanners by connecting community‐generated evidence, civic food networks and institutional partners to strengthen local food democracy and policy readiness. In line with collaborative (green) leadership scholarship, the work illustrates how dietitians convene stakeholders, manage tensions between health and sustainability aims, and leverage organisational platforms to align actions with net‐zero and equity agendas [10, 11]. These roles map directly to contemporary professional standards and that embed sustainability across dietetics practice domains [8, 9, 55].

Anchored in Ottawa Charter action areas and a socioecological framing, the programme produced shared measurements (i.e., diet affordability, food insecurity, retail healthiness, civic assets) that partners could jointly interpret. These data enabled development of a common agenda, leading to mutually reinforcing activities, and continuous communication within the Taskforce, with the federally‐funded Regional Development Australia Illawarra Shoalhaven acting as the backbone organisation, thus addressing the five core collective‐impact conditions [26].

Importantly, this regional food systems project demonstrates that dietitians can lead the assembly of shared measurement systems that translate community‐level evidence into governance‐ready artefacts (e.g., evidence briefs, food charter, action plan), to advocate for policy uptake. Results identified a dual reality whereby an obesogenic retail environment coexists with civic assets aligned with an alternative food network (including community and home food gardens, co‐ops and peri‐urban producers) that can be mobilised to improve access, build social capital, and shorten food supply chains. Locally relevant evidence allowed the Taskforce to target planning levers (zoning, procurement preferences) and highlight priorities for investment in community infrastructure.

Dietitian‐led teams can balance structural reform (planning, procurement, social protection) with community‐driven practice (literacy, civic production, co‐ops), avoiding over‐reliance on either charitable relief or education‐only approaches. Embedding feedback loops and reflexive practice helped navigate tensions (ownership vs. action; reliance on few actors; funding fragility) and maintain momentum, from evidence building to chartering and action planning [37, 56]. Governance for food systems transformation benefits from explicit learning architectures, such as scheduled reflections and open data sharing, that dietitians are well placed to curate.

### 4.1 Implications for Dietetic Practice and Research

This project provides four key learnings for dietetics practice, namely: operationalising food systems thinking in practice; reframing the professional identity of dietitians; recognising interdependence and collective impact; and embedding reflexivity and learning loops. Applying learning loops in a regional food systems transformative research project means embedding iterative cycles of reflection, feedback and adaptation into how the project operates, collaborates and evolves over time [57]. These loops help ensure that knowledge gained through action and experimentation continually improves both understanding and practice. By using learning loops, the project becomes a living laboratory that continuously evolves through cycles of enquiry, action and reflection. Dietitians can draw from this project as a model for translating systems thinking into practice. Instead of addressing nutrition issues solely through education or clinical care, the approach embeds dietitians in multi‐layered system interventions that span policy, community engagement, education and environment redesign [8, 9]. This initiative redefines dietitians as food systems changemakers, encompassing roles beyond traditional practice. Dietitians can fulfil many roles such as being knowledge translators (connecting scientific evidence with policy and public understanding), equity champions (ensuring that health and sustainability transitions are socially just) and disruptors and innovators (challenging entrenched norms within food environments and supply systems) [57]. These expanded roles align with the systems thinking principle that change occurs through interconnected agent networks, not isolated expertise [58].

Previous research has identified a perceived lack of social licence among nutrition professionals and dietitians to engage in food sustainability and broader food system action, despite growing recognition of the relevance of these domains to nutrition practice [59]. The findings of this case study suggest that social licence may be less an individual attribute and more an emergent implication of systems‐oriented, place‐based practice. By adopting roles as facilitators, knowledge translators and equity advocates, dietitians in this programme contributed to the co‐creation of a shared evidence base, governance structures and collective priorities. These processes may support the ongoing development of trust, legitimacy and acceptance across sectors, enabling dietitians to act within food system governance without overstepping perceived professional boundaries [60].

Systems thinking highlights that no single actor can transform a food system alone and confirms the need for distributed agency. Dietitians can use their cross‐sector credibility to facilitate collaboration between government, producers, educators and consumers [9] and thereby act as boundary spanners who bridge technical knowledge and community realities [61]. This project has demonstrated that systems transformation is dynamic, requiring continuous reflection and adaptation. Dietitians can apply this by using participatory evaluation frameworks and feedback mechanisms to refine interventions as new insights emerge.

To prepare future dietitians and nutrition professionals to address the urgent challenge of producing food that supports both human and planetary health, dietetics curricula need to expand to include competencies for food systems change [5, 57]. Current education often overlooks critical topics such as agriculture, ecology, biodiversity, climate change science, sustainability, economics and community development—areas essential for understanding the broader environmental and social contexts of nutrition [62]. By integrating these subjects into university programmes, educators can cultivate systems thinking, empowering students to engage in sustainable food production and policy advocacy. Moreover, embedding these skills lays the foundation for lifelong learning, ensuring practitioners continue to adapt as global food systems evolve within finite planetary boundaries.

## 5 Conclusion

Adopting systems thinking shifts the focus from individual‐level nutrition change to structural transformation of food environments and policies. A dietitian‐led, evidence‐driven, Collective Impact approach has demonstrated that diverse empirical inputs can be translated into regional food governance, shared agendas and actionable plans, offering a replicable pathway for municipal and regional food‐policy transformation. For the dietetics profession to be considered integral to food systems change will require integration of systems mapping and complexity analysis into university curricula and continuous professional development programmes, building partnerships with regional development organisations, local councils and community networks to co‐create interventions, and measuring outcomes not only in dietary behaviour but also in equity, resilience and ecological sustainability. This case from the Illawarra Shoalhaven region in Australia models how dietitians can activate systems change at regional and national scales by linking food security, planetary health and community wellbeing into a coherent, actionable framework.

## Author Contributions

Study conceptualisation: Karen Charlton and Katherine Kent. Data acquisition: Karen Charlton, Katherine Kent, Alemayehu Digssie Gebremariam, Suzanne Pickles and Anita Stefoska‐Needham. Data analysis: Karen Charlton, Karen Charlton, Katherine Kent, Alemayehu Digssie Gebremariam, Suzanne Pickles and Anita Stefoska‐Needham. Interpretation: Karen Charlton, Katherine Kent, Alemayehu Digssie Gebremariam, Suzanne Pickles, Anita Stefoska‐Needham and Selena Stevens. Writing of original manuscript: Karen Charlton. Editing: Karen Charlton, Katherine Kent, Alemayehu Digssie Gebremariam, Suzanne Pickles, Anita Stefoska‐Needham and Selena Stevens.

## Ethics Statement

‘Creating Future Food Warriors’: Providing experiential learning opportunities to address urban agriculture. UOW Human Research Ethics Committee (HREC). 2024/153. Veggie Matters: A pilot study to improve diet quality, nutritional status and perceptions about food. UOW Human Research Ethics Committee (HREC). 2024/258. ‘Let's talk about food….’: Cross‐sectional surveys exploring food security, food access, diet quality. UOW Human Research Ethics Committee (HREC)2024/030. Assessing the Impact of Engagement with Farmwall Vertical Gardens on University Students' Attitudes. UOW Human Research Ethics Committee (HREC). 2024/024. Exploring community, commercial and home urban agriculture initiatives across the Illawarra Shoalhaven. UOW Human Research Ethics Committee (HREC). 2023/217. Developing an Action Plan for a local sustainable, healthy and equitable food system in the Illawarra and Shoalhaven: UOW Human Research Ethics Committee (HREC). 2025/255 Social Network Analysis of key actors and their relationships in the Illawarra Shoalhaven food system: UOW Human Research Ethics Committee (HREC) 2024/330.

## Conflicts of Interest

The authors declare no conflicts of interest.

## Supporting information

Supporting Table

## Data Availability

The data that support the findings of this study are available on request from the corresponding author. The data are not publicly available due to privacy or ethical restrictions.
