# Supplementary material for: Dietitians as Food Systems Changemakers: The Path to Developing a Resilient, Equitable, Healthy and Connected Food System in the Illawarra Shoalhaven Region of Australia
Source: J Hum Nutr Diet. 2026 May 6;39:e70264. doi: 10.1111/jhn.70264 (PMC13147489; doi:10.1111/jhn.70264)
Supplement: Supplementary file 1 — Supporting Table [file JHN-39-0-s001.docx]

| Authors | Title | Date | Access link | Views | Down-loads |
| --- | --- | --- | --- | --- | --- |
| Andy Zhao, [Katherine Kent](https://ro.uow.edu.au/authors/Katherine_Kent/19586251), [Karen Charlton](https://ro.uow.edu.au/authors/Karen_Charlton/19585450) | Are school canteens in the Illawarra and Shoalhaven set up for universal school meal programs? | 13/11/2025 | <https://doi.org/10.71747/uow-r3gk326m.30606629> | 89 | 20 |
| [Karen Charlton](https://ro.uow.edu.au/authors/Karen_Charlton/19585450), [Katherine Kent](https://ro.uow.edu.au/authors/Katherine_Kent/19586251), Alemayehu Gebremariam | Shellharbour’s food environment | 2024-11-19 | https://ro.uow.edu.au/smhpapers1/1820 | 484 | 117 |
| [Karen Charlton](https://ro.uow.edu.au/authors/Karen_Charlton/19585450), [Katherine Kent](https://ro.uow.edu.au/authors/Katherine_Kent/19586251), [Alemayehu Gebremariam](https://ro.uow.edu.au/authors/Alemayehu_Gebremariam/19575244) | Shoalhaven’s food environment | 2024-11-18 | <https://hdl.handle.net/10779/uow.27813675> | 486 | 131 |
| [Karen Charlton](https://ro.uow.edu.au/authors/Karen_Charlton/19585450), [Katherine Kent](https://ro.uow.edu.au/authors/Katherine_Kent/19586251), [Alemayehu Gebremariam](https://ro.uow.edu.au/authors/Alemayehu_Gebremariam/19575244) | Kiama’s food environment | 2024-11-18 | <https://hdl.handle.net/10779/uow.27813672> | 419 | 184 |
| [Karen Charlton](https://ro.uow.edu.au/authors/Karen_Charlton/19585450), [Katherine Kent](https://ro.uow.edu.au/authors/Katherine_Kent/19586251), [Alemayehu Gebremariam](https://ro.uow.edu.au/authors/Alemayehu_Gebremariam/19575244) | Wollongong’s food environment | 2024-11-18 | <https://hdl.handle.net/10779/uow.27813669> | 552 | 112 |
| [Karen Charlton](https://ro.uow.edu.au/authors/Karen_Charlton/19585450), [Katherine Kent](https://ro.uow.edu.au/authors/Katherine_Kent/19586251), [Alemayehu Gebremariam](https://ro.uow.edu.au/authors/Alemayehu_Gebremariam/19575244) | Illawarra and Shoalhaven region’s food environment | 2024-11-18 | <https://hdl.handle.net/10779/uow.27813663> | 1004 | 286 |
| [Karen Charlton](https://ro.uow.edu.au/authors/Karen_Charlton/19585450), [Katherine Kent](https://ro.uow.edu.au/authors/Katherine_Kent/19586251), [Alemayehu Gebremariam](https://ro.uow.edu.au/authors/Alemayehu_Gebremariam/19575244) | Europbodalla Shire’s food environment | 2024-11-18 | <https://hdl.handle.net/10779/uow.27813642> | 390 | 146 |
| [Karen Charlton](https://ro.uow.edu.au/authors/Karen_Charlton/19585450), [Katherine Kent](https://ro.uow.edu.au/authors/Katherine_Kent/19586251), [Alemayehu Gebremariam](https://ro.uow.edu.au/authors/Alemayehu_Gebremariam/19575244) | Bega Valley’s food environment | 2024-11-18 | <https://hdl.handle.net/10779/uow.27813639> | 432 | 125 |
| Katy Fishlock, [Karen Charlton](https://ro.uow.edu.au/authors/Karen_Charlton/19585450), [Karen Walton](https://ro.uow.edu.au/authors/Karen_Walton/19582882), [Katherine Kent](https://ro.uow.edu.au/authors/Katherine_Kent/19586251), Meron Lewis | How Much Does It Cost to Buy Healthy Food in the Illawarra? | 2024-11-18 | <https://hdl.handle.net/10779/uow.27813654> | 2742* | 272 |
| Sammy Dean, Meron Lewis, [Karen Walton](https://ro.uow.edu.au/authors/Karen_Walton/19582882), [Katherine Kent](https://ro.uow.edu.au/authors/Katherine_Kent/19586251), [Karen Charlton](https://ro.uow.edu.au/authors/Karen_Charlton/19585450) | How Much Does It Cost to Buy Healthy Food in the Fowler Electorate, NSW? | 2024-11-18 | <https://hdl.handle.net/10779/uow.27813636> | 447 | 242 |
| [Katherine Kent](https://ro.uow.edu.au/authors/Katherine_Kent/19586251), [Karen Charlton](https://ro.uow.edu.au/authors/Karen_Charlton/19585450), Kelly Andrews, Grace Potter | Food Relief in Focus: A Snapshot of Food Relief in the Illawarra and Shoalhaven – 2025 | 2025-08-27 | <https://doi.org/10.71747/uow-r3gk326m.29992381> | 312 | 40 |
| [Katherine Kent](https://ro.uow.edu.au/authors/Katherine_Kent/19586251), Karen Charlton, Kelly Andrews, Grace Potter | “Food is the Glue”: Community Centres in the Illawarra and Shoalhaven Use Food as a Gateway to Supporting Wellbeing & Connection | 2025-02-28 | <https://doi.org/10.71747/uow-r3gk326m.28509653> | 1858 | 210 |
| [Katherine Kent](https://ro.uow.edu.au/authors/Katherine_Kent/19586251), Karen Charlton, Kelly Andrews, Grace Potter | The Impact of Rising Cost of Food on Shopping and Eating in the Illawarra and Shoalhaven | 2025-02-28 | <https://doi.org/10.71747/uow-r3gk326m.28509614> | 1383 | 254 |
| [Katherine Kent](https://ro.uow.edu.au/authors/Katherine_Kent/19586251), Karen Charlton, Kelly Andrews, Grace Potter | Food Insecurity in the Illawarra and Shoalhaven | 2025-02-28 | <https://doi.org/10.71747/uow-r3gk326m.28509581> | 1889 | 195 |
| [Katherine Kent](https://ro.uow.edu.au/authors/Katherine_Kent/19586251), Karen Charlton, Alemayehu Gebremariam, Kelly Andrews, Grace Potter | Which Suburbs Have the Best Access to Affordable, Healthy Food in Illawarra and Shoalhaven? | 025-02-28 | <https://doi.org/10.71747/uow-r3gk326m.28509638> | 1179 | 216 |
| [Katherine Kent](https://ro.uow.edu.au/authors/Katherine_Kent/19586251), Suzanne Pickles, Karen Charlton, Kelly Andrews, Grace Potter | Community Perspectives on Local Food in the Illawarra and Shoalhaven | 2025-02-28 | <https://doi.org/10.71747/uow-r3gk326m.28509626> | 1163 | 251 |
| [Suzanne Pickles](https://ro.uow.edu.au/authors/Suzanne_Pickles/19579093), [Karen 757Charlton](https://ro.uow.edu.au/authors/Karen_Charlton/19585450), [Anne-Therese McMahon](https://ro.uow.edu.au/authors/Anne-Therese_McMahon/19584940), [Anita Stefoska-Needham](https://ro.uow.edu.au/authors/Anita_Stefoska-Needham/19582567), [Katherine Kent](https://ro.uow.edu.au/authors/Katherine_Kent/19586251) | Urban Agriculture in the Illawarra and Shoalhaven: Community Gardens | 2024-11-18 | <https://hdl.handle.net/10779/uow.27813651> | 751 | 190 |
| [Suzanne Pickles](https://ro.uow.edu.au/authors/Suzanne_Pickles/19579093), [Karen Charlton](https://ro.uow.edu.au/authors/Karen_Charlton/19585450), [Anne-Therese McMahon](https://ro.uow.edu.au/authors/Anne-Therese_McMahon/19584940), [Anita Stefoska-Needham](https://ro.uow.edu.au/authors/Anita_Stefoska-Needham/19582567), [Katherine Kent](https://ro.uow.edu.au/authors/Katherine_Kent/19586251) | Urban Agriculture in the Illawarra and Shoalhaven: Commercial and Social Enterprises | 2024-11-18 | <https://hdl.handle.net/10779/uow.27813648> | 804 | 176 |

**Supplementary Table 1:** Impact metrics for publicly accessible evidence briefs that were disseminated through community partners

*Altmetrics = 32
